# Genome-Wide Identification and Characterization of *CPR5* Genes in *Gossypium* Reveals Their Potential Role in Trichome Development

**DOI:** 10.3389/fgene.2022.921096

**Published:** 2022-06-08

**Authors:** Heng Wang, Muhammad Jawad Umer, Fang Liu, Xiaoyan Cai, Jie Zheng, Yanchao Xu, Yuqing Hou, Zhongli Zhou

**Affiliations:** ^1^ State Key Laboratory of Cotton Biology /Institute of Cotton Research, Chinese Academy of Agricultural Science, Anyang, China; ^2^ School of Agricultural Sciences, Zhengzhou University, Zhengzhou, China; ^3^ National Nanfan Research Institute (Sanya), Chinese Academy of Agriculture Sciences, Sanya, China; ^4^ Hainan Yazhou Bay Seed Laboratory, Sanya, China; ^5^ College of Plant Science and Technology, Huazhong Agricultural University, Wuhan, China

**Keywords:** cotton, *CPR5* genes, co-expression network analysis, gene expression, trichome development

## Abstract

Trichomes protect plants against insects, microbes, herbivores, and abiotic damages and assist seed dispersal. The function of *CPR5* genes have been found to be involved in the trichome development but the research on the underlying genetic and molecular mechanisms are extremely limited. Herein, genome wide identification and characterization of *CPR5* genes was performed. In total, 26 *CPR5* family members were identified in Gossypium species. Phylogenetic analysis, structural characteristics, and synteny analysis of *CPR5*s showed the conserved evolution relationships of *CPR5*. The promoter analysis of *CPR5* genes revealed hormone, stress, and development-related cis-elements. Gene ontology (GO) enrichment analysis showed that the *CPR5* genes were largely related to biological regulation, developmental process, multicellular organismal process. Protein-protein interaction analysis predicted several trichome development related proteins (SIM, LGO, and GRL) directly interacting with *CPR5* genes. Further, nine putative *Gossypium*-miRNAs were also identified, targeting *Gossypium CPR5* genes. RNA-Seq data of *G. arboreum* (with trichomes) and *G. herbaceum* (with no trichomes) was used to perform the co-expression network analysis. *GheCPR5.1* was identified as a hub gene in a co-expression network analysis. RT-qPCR of *GheCPR5.1* gene in different tissues suggests that this gene has higher expressions in the petiole and might be a key candidate involved in the trichome development. Virus induced gene silencing of *GheCPR5.1 (Ghe02G17590)* confirms its role in trichome development and elongation. Current results provide proofs of the possible role of *CPR5* genes and provide preliminary information for further studies of *GheCPR5.1* functions in trichome development.

## Introduction

Trichomes have been found in most terrestrial plants and been proved to be the first barrier to protect from insects while being harmless to the environment. (Konarska and Lotocka, 2020; Kitagawa and Jackson, 2022). The special structure of trichomes, which are generally unicellular and multicellular, make trichomes a unique model to study the cell development process on plants (Kusstatscher et al., 2020; Ghazalli et al., 2021). During the past decades, the research on the formation mechanism of plant trichomes showed that multiple genes participate in the regulation of trichome initiation and elongation, such as genes involved with classical *bHLH-MYB-WD40* complex (Liu et al., 2021; Li et al., 2022). However, various genes which also play important roles in trichome formation had been not studied deeply, such as *CPR5* (constitutive expressor of pathogenesis-related genes 5) (Bowling et al., 1997).

Cotton trichomes are mainly distributed on the surface of the seed, leaf, and stem. The trichomes located on the seed coat of cotton, also known as cotton fiber, are the main economic harvest product. Cotton leaf and stem surface trichomes are similar to cotton fiber in structure, and the development process is regulated by genes related to fiber development (Bao and Hua, 2014; Wan et al., 2014). *GhTCP14* was mainly expressed in cotton fiber, especially during the initiation and elongation of cotton fiber cells. When *GhTCP14* was overexpressed in *Arabidopsis thaliana*, the number and length of trichomes were significantly increased. Overexpressed *Arabidopsis thaliana* plants having *GhPIN1a, GhPIN6*, and *GhPIN8* had more and longer epidermal trichomes on the leaves (Zhang et al., 2017). *GbML1* and *GhMYB25*, related to fiber development, shared a similar expression pattern and function on trichomes density and length in *Arabidopsis thaliana* (Zhang et al., 2010). *GbML1* and *GhMYB25* form a physical interaction through the START-domain (*GbML1*) and SAD-domain (*GhMYB25*) (Walford et al., 2012). Therefore, it was speculated that *GbML1* may act as a molecular chaperone to regulate the development of fibroblasts by enhancing the expression of *GhMYB25. GaHOX1* of *Gossypium hirsutum* had high homology with *Gl2*, which was mainly expressed in fiber at the early stage of development (Wang et al., 2013). *GaHOX1* could complement the phenotypic defects of *Arabidopsis* mutant Gl2 and make it grow again. Overexpression of *GhHOX3* in *Gossypium hirsutum* could make the cotton fiber longer and silencing the gene could lead to a reduction of more than 80% of the cotton fiber length (Hu et al., 2018). There is a similarity between the mechanism of hairiness control and cotton fiber development. The research on the mechanism of trichome elongation and development can provide a reference for revealing the mechanism of cotton fiber development to a certain extent.

According to the gene function annotation, there were five genes involved with constitutive expression of pathogenesis-related (PR) genes, *CPR1* with F-box domain, *CPR5, CPR6, CPR20*, and *CPR21* (Wei et al., 2019; Anisimova et al., 2021). All of them had been detected as participating in signal transduction pathways involved in plant defense, but not sharing the same domain. Besides, only *CPR5* was found to relate with the trichome development process (Kirik et al., 2001; Peng et al., 2020), which made it more important to determine the resistance to biotic and abiotic stress in plant species.

*CPR5* was identified as a defense response gene with a striking feature 5’ C-terminal transmembrane segments (TMSs) in a 1 + 4 TMS arrangement (Van Dingenen, 2022). Generally, the TMSs structure of CPR5 protein C-terminal showed their function on being a nuclear membrane protein as a component of nuclear pore complex (NPC) and regulating the effector-triggered immunity (ETI) and programmed cell death (PCD) on plants (Yoshida et al., 2002; Peng et al., 2020). For the C-terminal parts of the *CPR5* protein, a previous study indicated that three parts existed: nuclear localization signals clusters, casein kinase phosphorylation sites clusters, and alternative mutation sites (Gu et al., 2016). *CPR5* participated in various pathways, including plant growth, bacterial and fungal resistance, and trichome development (Jing et al., 2007; Jing and Dijkwel, 2008).

According to past research, the *CPR5* was nearly found in plans, such as *Vitis vinifera*, *Theobroma cacao*, *Qryza sativa*, *Gossypium hirsutum*, and *Triticumm aestivum* (Orjuela et al., 2013; Pinel-Galzi et al., 2016). Various *CPR5* homologous genes were present in different plant species. The numbers of most *CPR5* genes were consistent with the genome numbers of the host plants. The *CPR5* gene was first cloned in an *Arabidopsis* mutant line which showed resistance to *Pseudomonas syringae* and abnormal trichomes (Kirik et al., 2001). Based on the research of *CPR5* sequence and structure, *CPR5* was found to be a transmembrane protein and sustain the balance between homeostasis, cell division, and cell death by gating multiple transcription factors entering the nucleus (Deuschle et al., 2001; Gao et al., 2011; Gu et al., 2016). The involvement of *CPR5* with plant disease defense had been deeply studied in *Arabidopsis*. The study showed that *CPR5* played important roles in signaling pathways, such as CKI-RB-E2F cell cycle signaling pathway, activated immune responses, and ROS signal transduction (Borghi et al., 2011; Chen et al., 2022). *CPR5* was also found to be involved with multiple plant hormones signaling pathways, such as salicylic acid and ethylene (Clarke et al., 2004). Besides the *CPR5* function to disease resistance, the mechanism of trichomes development also been detected in *CPR5* mutant plants, such as *Arabidopsis*, Soybean, and Mucuna (Okuma et al., 2014; Meng et al., 2017). The study showed that *CPR5* was a downstream gene controlled by two CKI signaling pathway genes, *SIM* and *SMR1*. *SIM SMR1* double loss mutant produced a cell death and branchless trichomes in Arabidopsis (Yi et al., 2014; Kumar et al., 2015). Another study showed that *UVI4* and *OSD1* interacted with *CPR5* to regulate the trichomes development, indicating that *CPR5* was essential in cell cycle progression (Heyman et al., 2011). Those studies had showed that *CPR5* was involved in trichomes development, but the mechanism was still unclear.

Currently, we performed the genome wide analysis of *CPR5* genes in cotton and explored their role in trichome development. Expression levels of the *CPR5* gene in different tissues suggests that *Ghe02G17590* might be the true candidate gene that plays a very important role in trichome development.

## Materials and Methods

### Identification of *CPR5* Gene Family Members From Cotton, *Arabidopsis thaliana*, *Oryza sativa*, *Theobroma cacao*, and *Vitis vinifera*

In order to identify *CPR5* genes, the sequences of one *At5g64930* protein was retrieved from the *A. thaliana* genome and was subsequently used for determining the *CPR5* genes in the genomes of *Gossypium arboreum* (A1), *Gossypium herbaceum* (A2), *Gossypium anomalum* (B1), *Gossypium sturtianum* (C1), *Gossypium thurberi* (D1), *Gossypium raimondii* (D5), *Gossypium stocksii* (E1), *Gossypium longicalyx* (F1), *Gossypium australe* (G2), *Gossypium rotundifolium* (K2), *Gossypium hirsutum* (AD1), *Gossypium barbadense* (AD2), *Gossypium tomentosum* (AD3), *Gossypium mustelinum* (AD4), *Gossypium darwinii* (AD5). Besides, four other plant species (*Arabidopsis thaliana, Oryza sativa, Theobroma cacao, Vitis vinifera*) *via* reciprocal blast with BLASTP program (Altschulet al., 1997). The default parameters with E-values of less than 1 E^−10^ were set in the BLASTP searches (Supplementary Table S1).

### Data Acquisition

The genome, protein, and structure information of *Gossypium herbaceum* (A1, version WHU_V1), *Gossypium arboretum* (A2, version WHU_updated_V1), *Gossypium raimondii* (D5, version JGI_V2_a2.1), *Gossypium hirsutum* (AD1, version HAU_V1.1), *Gossypium barbadense* (D5, version HAU_V2_a1), were downloaded form Cottongen (https://www.cottongen.org/). The data for the other ten cotton species were obtained from NCBI (https://www.ncbi.nlm.nih.gov/). Data for *rabidopsis thaliana, Oryza sativa, Theobroma cacao,* and *Vitis vinifera* were obtained from phytozome database 12 (https://phytozome-next.jgi.doe.gov/).

### Phylogenetic Analysis of *CPR5* Genes

The protein sequences of all the discovered CPR5 genes from the cotton species as well as *Arabidopsis thaliana*, *Oryza sativa*, *Theobroma cacao*, and *Vitis vinifera* were aligned *via* Clustal X using default parameters (Larkin et al., 2007). The phylogenetic tree of *CPR5* from each species was constructed using the numerous sequence alignments imported and displayed in MEGA seven using the neighbor joining approach. For statistical reliability, tree nodes were calculated using the Bootstrap method with 1,000 repeats (Tamura et al., 2007).

### Gene Structure and Chromosomal Location and Collinearity Analysis of *CPR5* Genes

The gene structure of *CPR5* genes was examined by GSDS 2.0 (https://gsds.cbi.pku.edu.cn/) using the Genomic DNA and CDS sequence of each species (Hu et al., 2015). CottonFGD genome annotation files were used to identify the chromosome locations of *CPR5* genes in these species. This information was also used to construct chromosomal mapping which were then displayed by TBtools (Chen et al., 2020). All of the proteins sequences were submitted to the online motif and domain identification tool MEME (http://meme-suite.org/) in order to discover the conserved domains present in the *CPR5* proteins (Bailey et al., 2009). The motif search was carried out using a total count of 15 motifs. The MAST tool was used to show the protein database for the identified motifs. Multicollinearity ScanToolkit was used for analyzing the synteny relationship between and within four cotton species, *G. hirsutum and G. barbadense, G. herbaceum*, and *G. raimondii*. The collinearity among orthologs and paralogs genes were displayed by TBtools (Chen et al., 2020).

### Subcellular Localization Prediction

The protein sequences of *CPR5* genes were uploaded to WoLF PSORT online website (https://wolfpsort.hgc.jp) for subcellular localization prediction.

### Analysis of Cis-Regulatory Elements of *CPR5* Genes

The promoter regions up to 2000 bp upstream in the *CPR5* genes were downloaded from the cotton database (www.cottonfgd.org) and used to analyze potential cis-regulatory elements by the online tool PlantCARE (Lescot et al., 2002) (https://bioinformatics.psb.ugent.be/webtools/plantcare/html/).

### Transcriptome Profiling of *G. arboreum* and *G. herbaceum*

*G. arboreum* and *G. herbaceum* were used for the transcriptome sequencing. The RNA-Seq libraries were prepared by a previously described method (Wang et al., 2018), and a 1% agarose gel was used to check for contamination and degradation of RNA. First, the purity of RNA was analyzed using a Nano Photometer® spectrophotometer (IMPLEN, CA, United States). Next, estimating RNA concentration was performed using a Qubit® RNA Assay Kit and a Qubit® 2.0 Fluorometer (Life Technologies, CA, United States). Finally, RNA integrity was checked using an Agilent Nano 6000 assay kit (Santa Clara, California, United States). Reads counting features (genes, in this case) were performed using HTSeq v0.6.125. Gene lengths and read counts mapped to genes were used to calculate FPKM values (Mortazavi et al., 2008). The original data was uploaded to NCBI (PRJNA833579).

The differentially expressed genes (DEGs) between diploid and tetraploid were identified with the DESeq R package (Andino et al., 2016), and Benjamini–Hochberg-adjusted *p*-values < 0.05 were considered statistically significant (Benjamini and Hochberg, 1995; Anders and Huber, 2010).

### Gene Ontology and Protein-Protein Network Analysis of *CPR5*

The gene ontology information of all *CPR5s* was obtained by using online genome-wide functional annotation tool EGGNOG-MAPPER (http://eggnog-mapper.embl.de/) and displayed by TBtools. The amino acid sequences of *Arabidopsis CPR5* genes were used as query sequences to obtain the protein-protein network by using STRING website (https://cn.string-db.org/).

### Prediction of Putative miRNA Targeting Gossypium *CPR5* Genes

The gene sequences of the *Gossypium CPR5* genes were used as candidate genes to identify possible miRNAs *via* observing against the existing Gossypium reference of miRNA sequences *via* the psRNATarget database (https://www.zhaolab.org/psRNATarget/analysis?function=2, accessed on 5 May 2022) with default parameters (Raza et al., 2021). Cytoscape (V3.8.2, https://cytoscape.org/download.html, accessed on 5 May 2022) software was used to build an interaction network between the identified miRNAs and the equivalent target *Gossypium CPR5* genes.

### Coexpression Network Analysis of *CPR5* Genes

We used RNA-Seq data of *G. arboreum* with trichomes and *G. herbaceum* with no trichomes to study the expression patterns of the *CPR5* gene family. The links between genes involved in trichome development were examined by coexpression network analysis. A coexpression regulation network was created using the Cytoscape software (version 3.7.2) (Shannon et al., 2003). The threshold for the coexpression network map was set as *p* > 0.99. The topological coefficient of each node with a degree >20 was used to identify the network as hub genes.

### Virus Induced Gene Silencing of *GheCPR5.1*

A 285 bp fragment from the CDS sequence of *GheCPR5.1* was selected to detect the gene function in regulation of trichome development. The TRV: 00 plasmid was digested with the restriction enzymes Eco*RI* and Bam*HI* and combined with the target fragment to generate TRV: *GheCPR5.1*. TRV: *GhCHL1* was used as the positive control. TRV: 00, TRV: *GheCPR5.1*, TRV: *GhCHL1* were transformed in *Agrobacterium tunefaciens* LBA4404 and infiltrated into the cotyledons of 10-day-old Dianya-10. Three biological replicates (each with 20 plants) were performed. Cotton seedlings were grown in the plastic pot filled with solid culture medium (vol/vol, sterile sand: vermiculite: nutritious soil = 1:1:2) in incubators at 25°C during the day and 20°C at night, with 60% relative humidity and a 16/8 h light/dark photoperiod. The leaf blenching phenotype appeared in the TRV: *GhCHL1* plants at 15–25 days, the trichome phenotype appeared in wild type plants at 20 days, we selected 25 days as the time to investigate the phenotype and VIGS silencing efficiency. The primers used for vector construction and RT-qPCR are listed in Supplementary Table S2. The number of trichomes present of the petiole of wild type, TRV: 00 and TRV: *GheCPR5.1* was counted under a Stereo microscope and classified as either villi (trichomes are extremely short and prostrate) or hairs (trichomes are long and upright). For each individual, the numbers of hair type trichomes were counted. The number of trichomes will be only counted on one side of petiole. The data was analyzed and displayed by Graphpad prism seven software.

### RT-qPCR Assay and Expression Analysis of *CPR5* Genes

The gene expression profiles of *GheCPR5.1* were analyzed from RNA-Seq data. The FPKM values were used to present the *GheCPR5.1*expression levels. RNA was extracted by TIANGEN kit following the protocol guidelines and was reverse transcribed into cDNA-by-cDNA synthesis SuperMix for qPCR (one step for gDNA removal). Finally, quantitative RT-qPCR was analyzed using the SYBR Green SuperMix kit according to the instruction manual. The experiment was performed using three technical and biological replications. The relative expression data were calculated using the 2^−ΔΔCT^ method (Schmittgen and Livak, 2008). The primers used in the experiment are designed *via* NCBI (https://www.ncbi.nlm.nih.gov/) and *Ghactin7* (*LOC107959437*) gene was used as an internal control (Supplementary Table S2).

## Results

### Identification of *CPR5* Genes

In total, 33 *CPR5* family members were detected in the plant species mentioned before four *CPR5* family members were found in *Oryza sativa* and *Gossypium barbadense*, three *CPR5* family members were found in *Gossypium hirsutum*, *Gossypium mustelium* and *Gossypium darwinii*, two were found in *Gossypium tomentosum*, 1 was found in *Arabidopsis*, *Vitis vinifera*, *Theobroma cacao*, and all ten diploid cotton species, respectively. After filtering the splicing transcripts of *CPR5s*, the result showed that all tetraploid cotton species included only two *CPR5s*, including *O. sativa*. The tetraploid cotton species contain twice the number of *CPR5* genes as compared to diploid cotton. The result showed the conserved process of *CPR5* gene family duplication during cotton whole genome duplicated events. The *CPR5* gene family were renamed according to their positions on chromosomes and the number of splicing viraties. Ghe, Gar, Ghir, Gbar, Gtom, Gmus, Gdar, Gano, Gstu, Gthu. Grai, Gsto, Glon, Gaus, and Grot, were used as prefixes before the names of *CPR5* genes, respectively.

Among all 26 *CPR5* genes in Gossypium, the amino acid length ranged from 401 amino acid (*G. hirsutum* and *G. barbadanse*) to 610 amino acid (*G. thurberii*). PI (isoelectric point) ranged from 8.56 (*GlonCPR5.1*) to 9.26 (*G.thurberi*), MW (molecular weight) ranged from 30379.1 (*GlonCPR5.1*) to 68467.08 (*GthuCPR5.1*), Instability index ranged from 37.26 (*GlonCPR5.2*) to 52.08 (*GtomCPR5.2*). Most of the *CPR5* genes were predicted to be located in the plasma membrane. Physical and chemical properties of *CPR5* genes in *Gossypium* were given in (Supplementary Table S1).

### Phylogenetic Analysis of *CPR5* Genes

**FIGURE 1:**
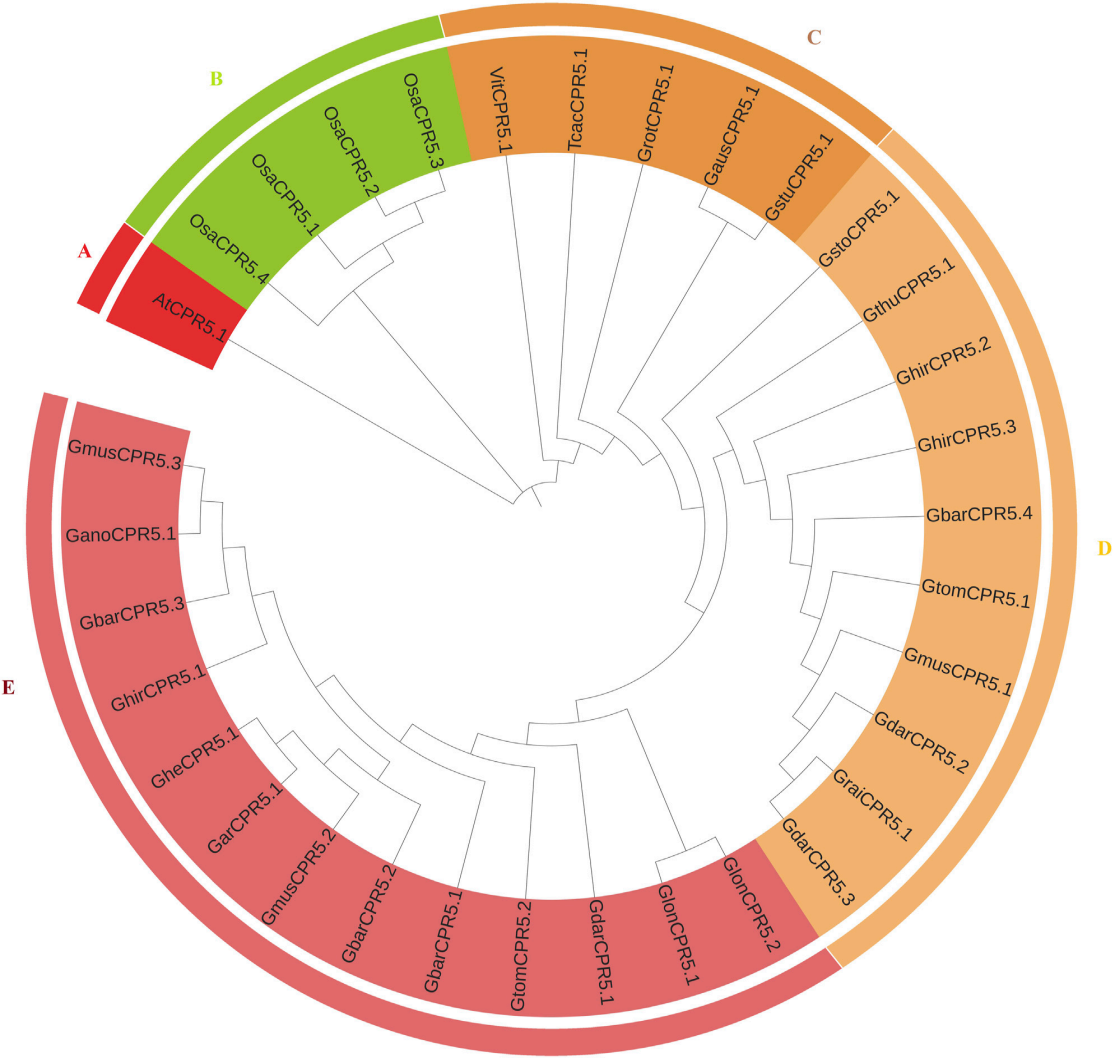
Phylogenetic tree of *CPR5* proteins from *A. thaliana*, *O. sativa, Vitis vinifera,* and *Theobroma cacao*. The *CPR5* were divided into five groups **(A–E)** on the clustering of the protein sequences. Cluster A contains one protein from *A. thaliana*, cluster B contains four proteins from *O. sativa*. Cluster C contains the proteins from *G. herbaceum*, *Gossypium mustelium, Gossypium darwinii*, *Gossypium tomentosum, Gossypium hirsutum, Gossypium sturtianum, Gossypium austral, Gossypium rotundifolium. T. cacao,* and *Vitis vinifera*, *whereas,* clades **(D,E)** contains 10 and 13 genes each belonging to Gossypium species.

To better understand the evolutionary relationship among *CPR5* genes in Gossypium species mentioned in materials part and *O. sativa, T. cacao, A. thaliana,* the amino acid sequences were aligned with Clustal X software, and an unrooted phylogenetic tree was constructed using MEGA seven software (Figure 1). For the phylogenetic tree among eleven plant species, the 33 *CPR5s* were divided into five clades (clade A to E) depending on the common conserved *CPR5* features. Clade E and D were the largest clades containing 13 and 10 *CPR5s* genes respectively and interestingly all of them belong to Gossypium species. Clade C with five *CPR5* genes showed more associations with *Vitis vinifera* and *Theobroma cacao.* Clade A contains only one *CPR5* gene belonging to Arabidopsis. Meanwhile clade B had four *CPR5* genes belonging to *O. sativa.* The results suggested that Gossypium *CPR5s* show a closer relationship with *CPR5s* from *Vitis vinifera* and *Theobroma cacao*. The phylogenetic analysis suggests that the cotton *CPR5* genes are more closely linked to each other. For example, pairs of homologous genes, *GheCPR5.1/GarCPR5.1*, *GhirCPR5.1/GbarCPR5.3* were clustered in one group.

### Gene Architecture and Motif Analysis

**FIGURE 2:**
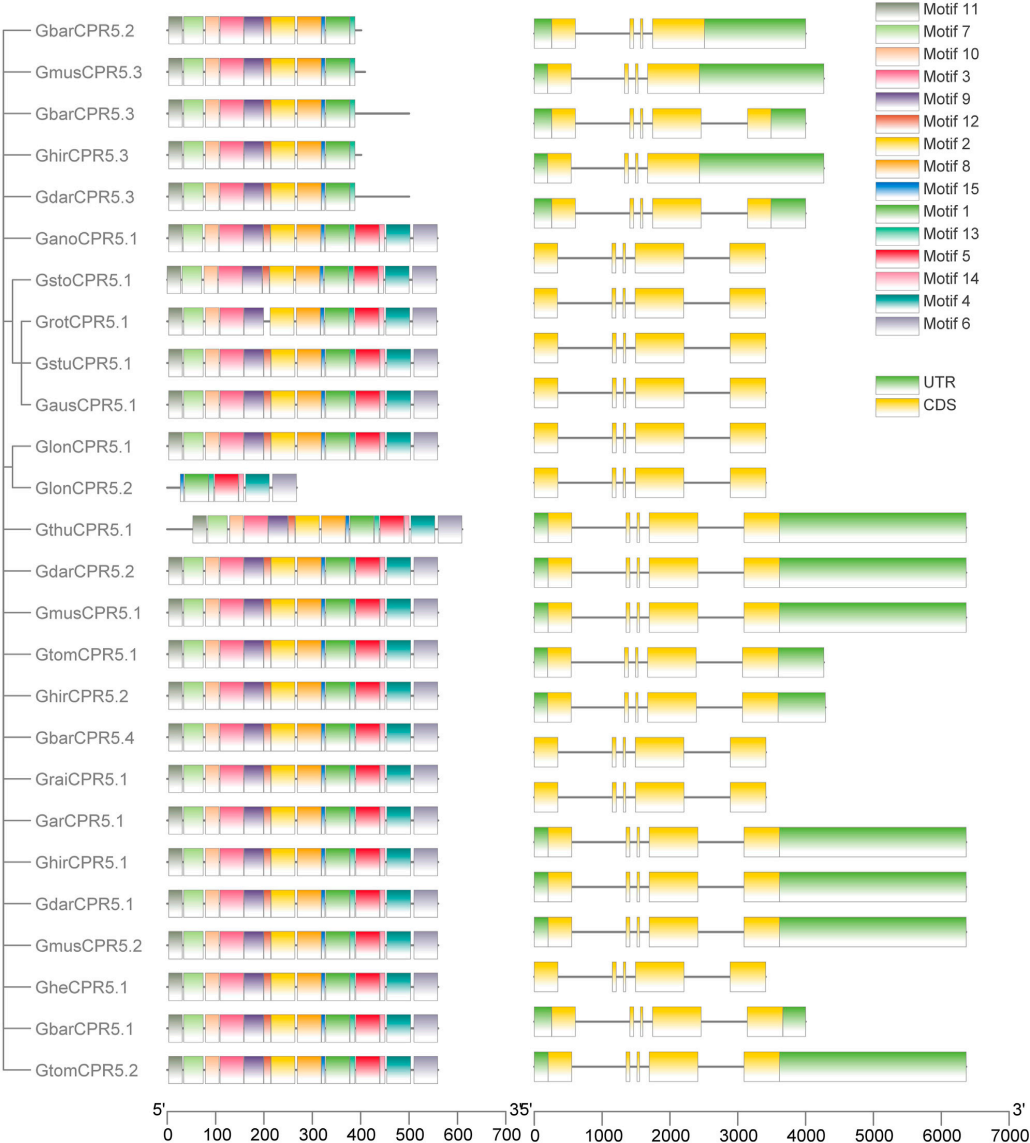
Motif and gene structure analysis of *CPR5* genes. On the left side is the distribution of the *CPR5* conserved motifs in cotton and rice. Five motifs, i.e., motifs 1, 2, 6, 7, and 13 were conserved motifs, shown by green, yellow, purple, light green, and magenta. Right side: intron and exon structure of cotton and rice *CPR5* genes. Exons and the UTR are shown as green and yellow boxes, respectively whereas introns are represented as grey lines.

We investigated the genetic architecture of *CPR5* proteins by studying the exon-intron structural distribution (Figure 2). The number and size of introns and exons of genes were usually conserved in plant species. The conserved features could be used to detect the evolutionary relationship among the gene family. The results showed that *CPR5s* of all Gossypium species which we used in this study harbored four introns and five exons except the variety splicing *CPR5* genes, such as *GbarCPR5.2* and *GhirCPR5.1.*

By analyzing the protein sequences of *CPR5* genes in Gossypium species using the MEME online tool, 15 motifs with significant E-values were found. The most five conserved motifs, motif 1, 2, 6, 7, and 13 were observed in all *CPR5s*. According to the *CPR5* motif function annotation in Arabidopsis, these five motifs were identified as five transmembrane features, which played an essential role in regulating multiple pathways of *CPR5*. Motifs 4, 10, 11, 12, and 14 were only found in Gossypium species. Those five motifs sites in Arabidopsis were predicted as Mobidblt-Consensus Disorder region, which was identified as containing putative nuclear localization signal sites. Most *CPR5* genes clustered in the same group shared the same motif features. For example, the *CPR5s* lacking motifs 2, 6, 7, and 13 were only observed in group 2. The result of motif analysis was consistent with the phylogenetic relationship. The clades with special motifs likely shared different functions.

### Chromosomal Mapping of *CPR5* Genes

**FIGURE 3:**
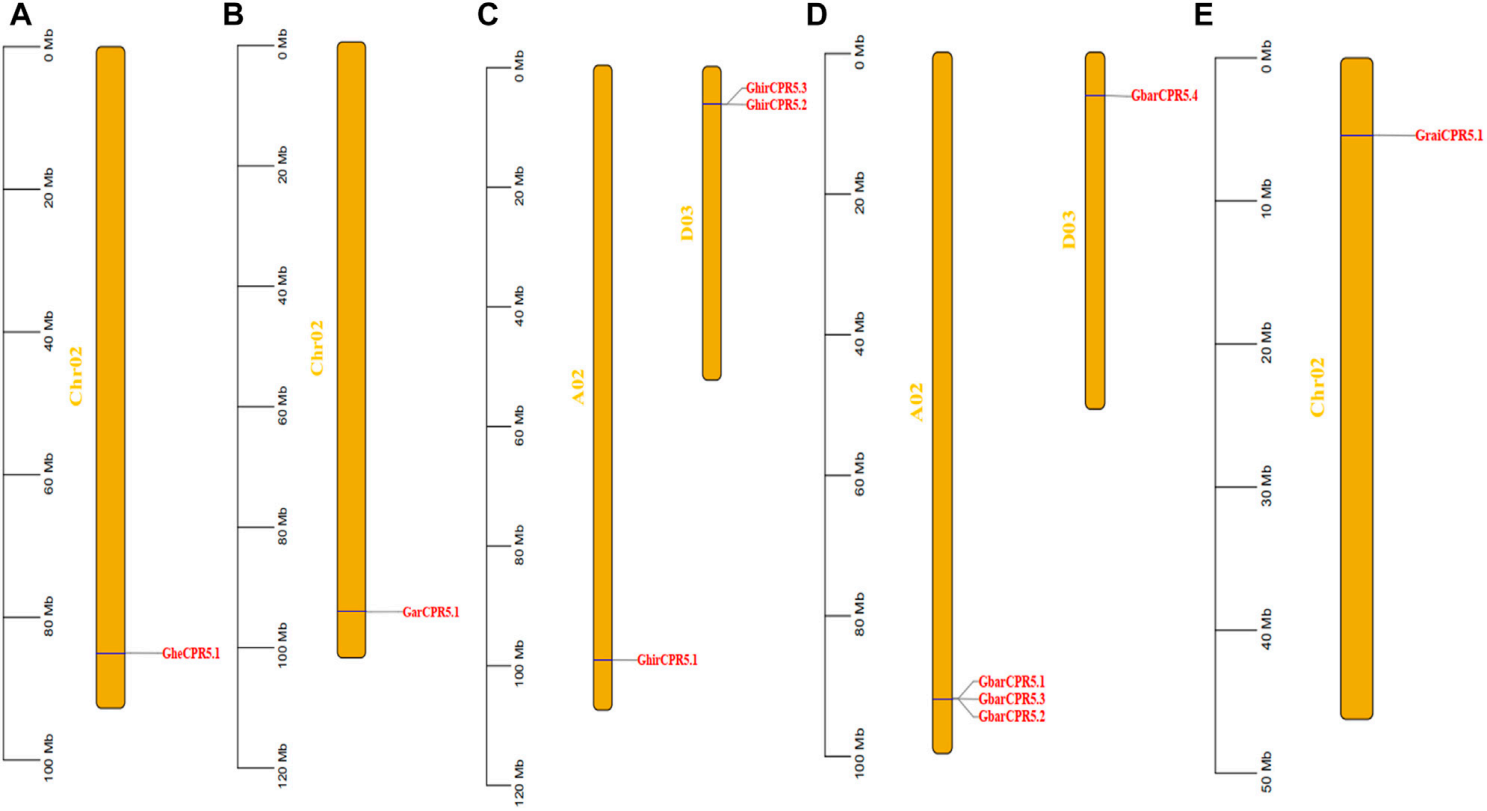
Distribution of the *CPR5* genes on chromosomes of different cotton species i.e., A1, A2, AD1, AD2, D5. **(A)** A1 (*Gossypium herbaceum*). **(B)** A2 (*Gossypium arboreum*). **(C)** AD1 (*Gossypium hirsutum*). **(D)** AD2 (*Gossypium barbadense*). **(E)** D5 (*Gossypium raimondii*). The scale bar represents the chromosome length. The yellow bar indicates the chromosomes. The gene on each chromosome is highlighted in red.

The gene distribution of *CPR5* genes on the chromosomes varies between the tetraploid and diploid cotton species (Figure 3). For diploid Gossypium species, *G. herbaceum, G. arboretum, and G. raimondii*, the *CPR5* genes were only found on chromosomes 2. For tetraploid Gossypium species, the *CPR5* genes were only found on chromosomes 2 and chromosomes 3. The gene distribution of *G. herbaceum, G. arboreum*, and *G. raimondii* was shown to be highly conserved and consistent with the A and D subgenome of *G. hirsutum* and *G. barbadense,* indicating the *CPR5s* conserved the evolution pattern between diploid cotton and tetraploid cotton. All *CPR5* genes were found on the two end sides of chromosomes in cotton.

### Evolutionary Relationship and Systemic Association of *CPR5s* in Cotton

**FIGURE 4:**
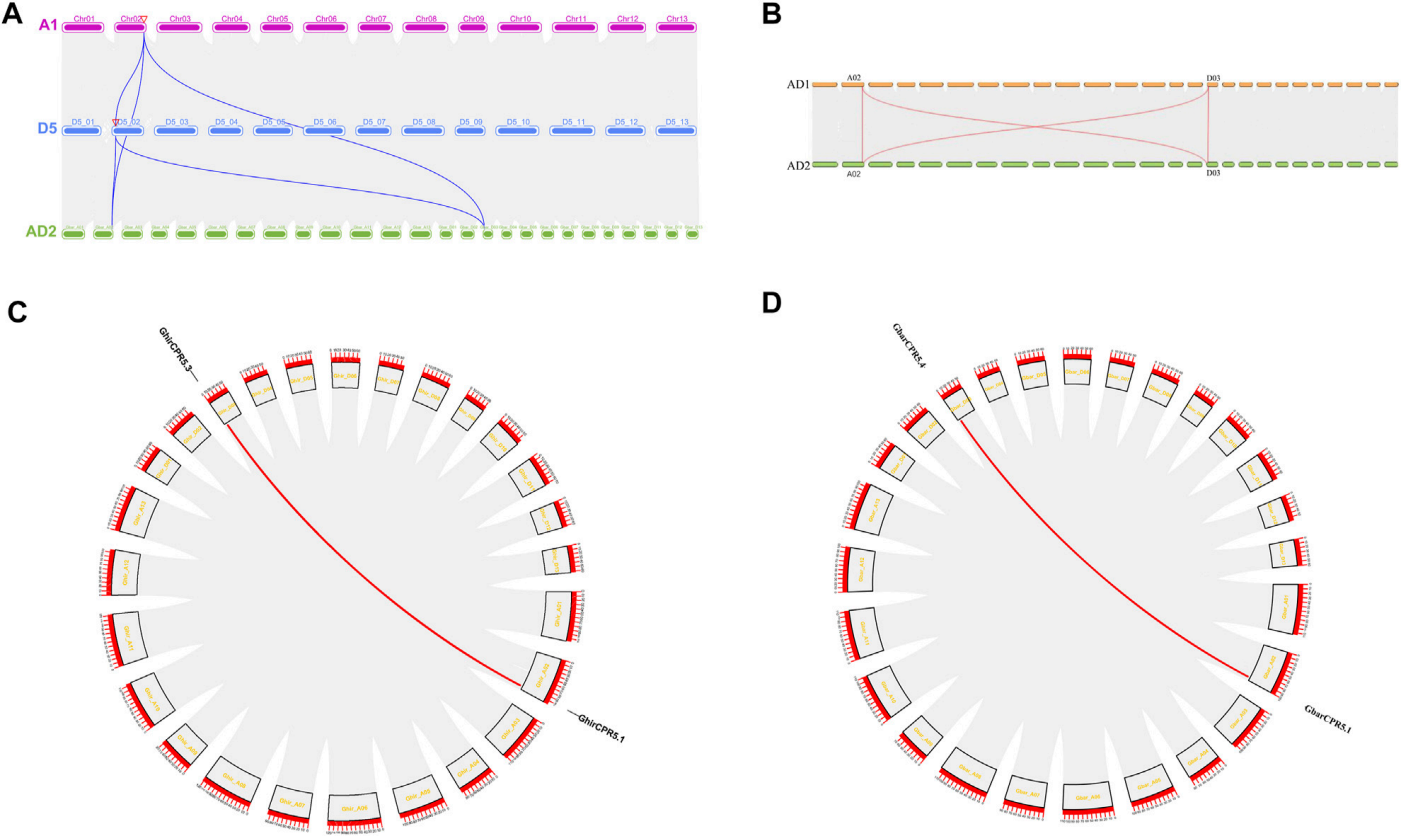
Syntenic relationships among CPR5 genes in A2, D5 and AD2 and AD1 and AD2. **(A, B)** *CPR5* genes are plotted against their projected complements in the five species. The chromosomes of AD1 are shown as number A1‐A13, and the chromosomes of D5 are shown as D2-01 to D2-13 whereas in AD2 the chromosomes were shown as Gbar A1-Gbar-A13. The grey lines indicate the collinear blocks between different species. The blue lines represent the collinear *CPR5* gene pairs between A2, D5 and AD2. The orange line represents the collinear *CPR5* gene pairs between AD1 and AD2. **(C, D)** The *CPR5* collinear gene pairs on A2, D5 and AD2 chromosomes. Red line represents a pair of collinear genes.

The gene duplication was the main reason to produce and expand the gene family. Three events were observed in plant species gene duplication, including tandem (a chromosomal region within 200 kb containing two or more genes is defined as a tandem duplication event), segmental (multiple genes through polyploidy followed by chromosome rearrangements), and whole genome duplication (the process by which a region of DNA coding for a gene creates additional copies of the gene). The gene duplication events may influence the gene function diversity among plant species. In this study, we used two tetraploid cotton species, *G. hirsutum* (AD1) and *G. barbadense* (AD2), and two diploid cotton species. *G. herbaceum* (A2) and *G. raimondii* (D5) to demonstrate the evolutionary relationship and syntenic association of *CPR5s* in cotton. Synteny analysis between *G. hirsutum* (AD1)*, G. herbaceum* (A2) and *G. raimondii* (D5) revealed that the *CPR5s* were reserved among three species (Figure 4). For within genome synteny analysis, two duplication gene pairs, *GhirCPR5.1/GhirCPR5.3* and *GbarCPR5.4/GbarCPR5.1*, were found in *G. hirsutum* and *G. barbadense*, respectively (Figures 4C,D).

### Cis-Regulatory Elements Analysis

**FIGURE 5:**
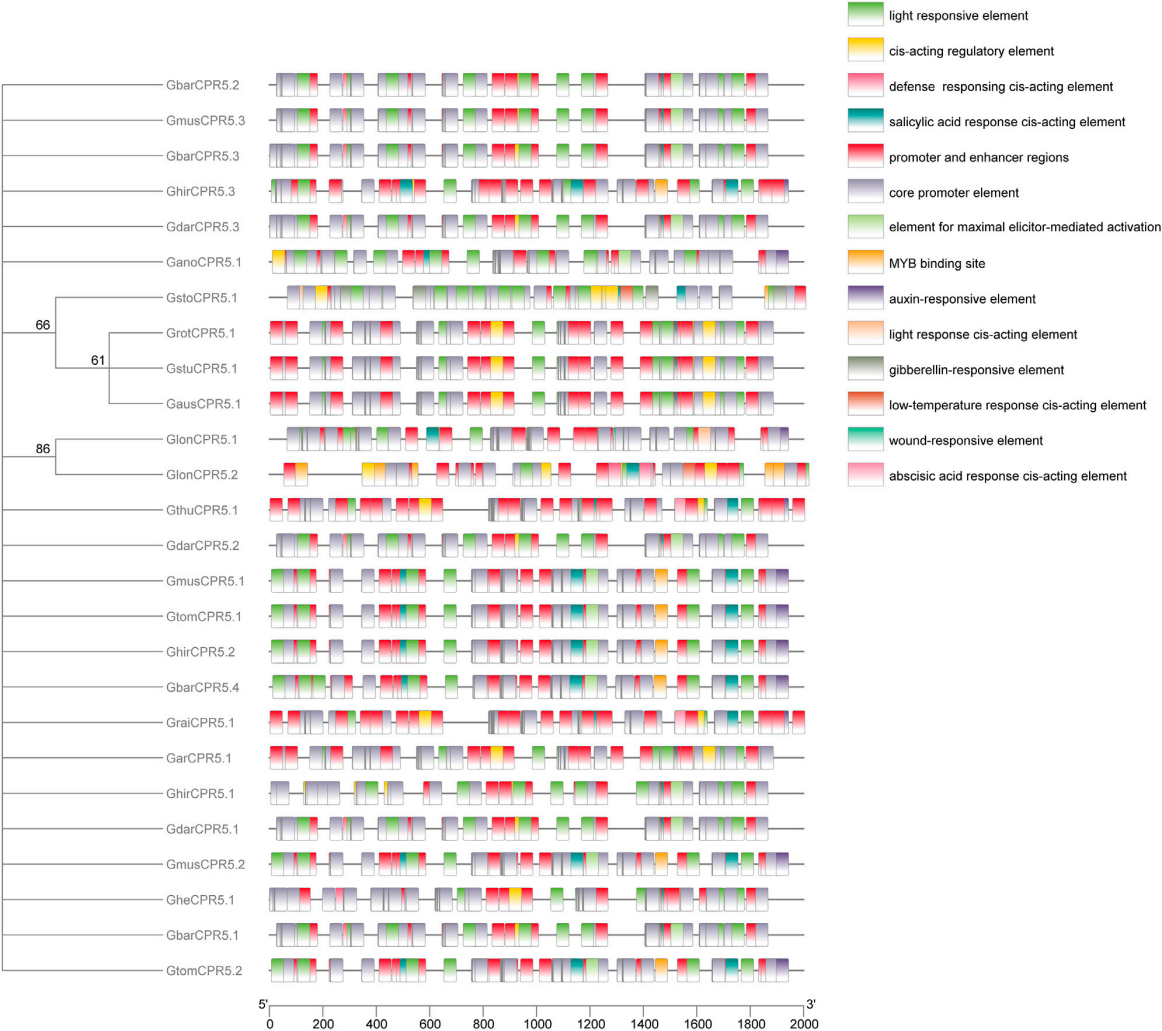
Predicted cis-elements in *CPR5* promoters. Promoter sequences (2,000 bp upstream regions) of 18 *CPR5* genes were examined online at PlantCARE web server. Diverse colors were used for representing different cis-elements, as given in on the right side.

The cis-elements analysis results showed that the TCA-element and TATA-box motifs were distributed in all *CPR5* genes of Gossypium species (Figure 5). Compared with other diploid cotton species, the number of TCA-elements and TATA-box motifs were higher in tetraploid cotton species. The results revealed that these two cis-elements have essential roles in *CPR5* function in disease resistance and stress response. All cis-elements predicted from cotton species are mostly involved in stress environment response, such as light response, low temperature, wound, and multiple hormone pathways, such as salicylic acid, abscisic acid, auxin, and gibberellin. Generally, the light response elements and salicylic acid response elements are distributed in most *CPR5s.*

### Gene Ontology and Protein-Protein Network Analysis of *CPR5*

**FIGURE 6:**
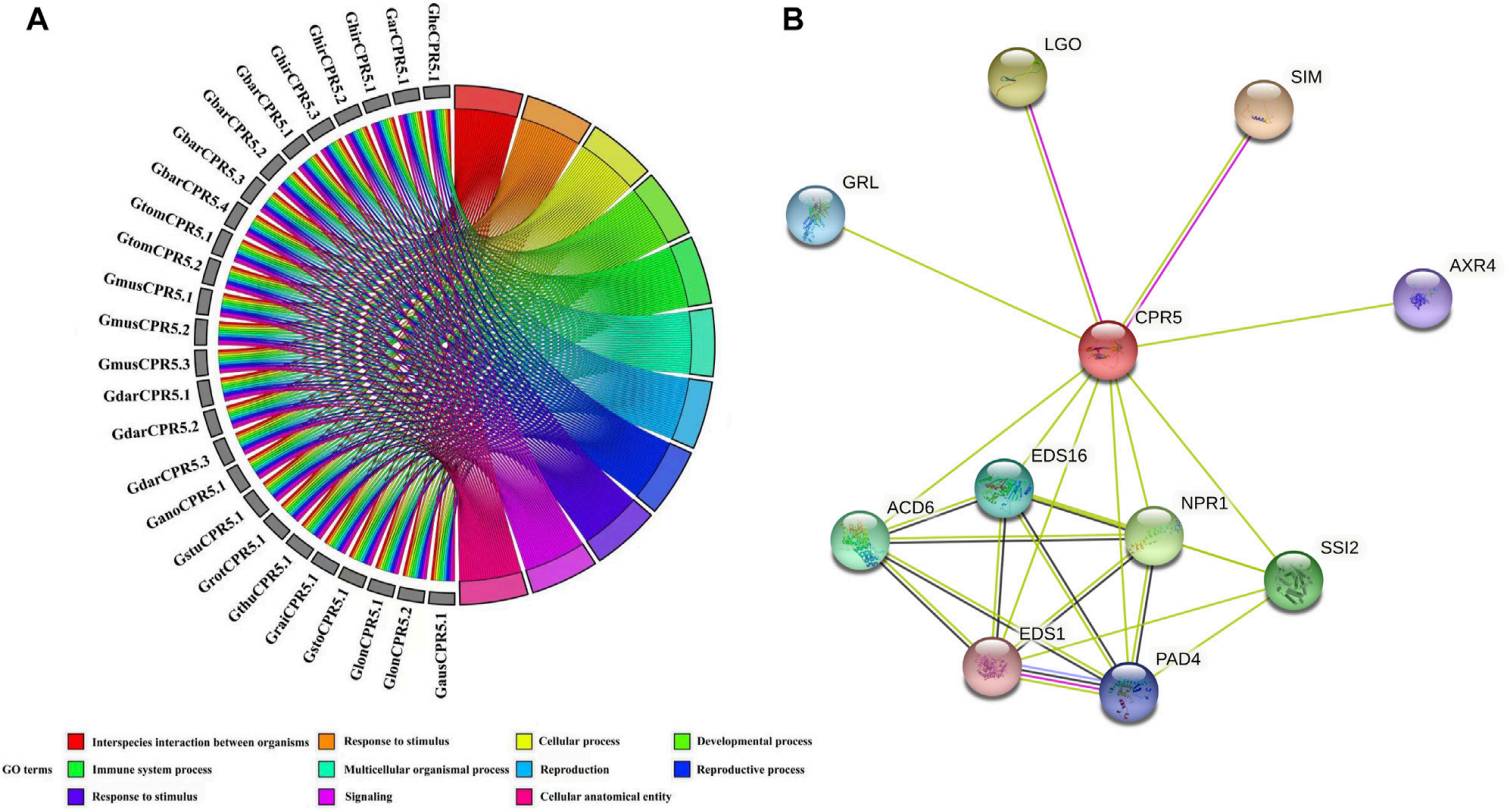
Gene ontology and protein-protein network analysis of *CPR5*. **(A)-** Gene ontology analysis of *CPR5* genes. **(B)-** Protein-protein analysis of *CPR5* in *Arabidopsis*. Colored nodes: query proteins and first shell of interactors. Red lines: gene fusions. Green lines: gene neighborhood. Black lines: co-expression. Blue lines: gene co-occurrence.

To identify the function of 26 *CPR5* family genes, we performed the GO annotation and enrichment analysis based on their biological process, molecular function, and cellular component classes. The GO annotation results showed that several GO terms were enriched in biological processes and cellular components (Figure 6A). The GO-biological process enrichment results suggested that 10 terms were highly enriched, such as biological process involved in interspecies interaction between organisms (GO: 0044419), biological regulation (GO: 0065007), developmental process (GO: 0032502), immune system process (GO: 0002376), multicellular organismal process (GO: 0032501), response to stimulus (GO: 0050896), etc., These terms confirmed the function of *CPR5* genes in the disease defense and immune system regulation of plants. The GO-cellular component enrichment results suggested that one term was highly enriched, the cellular anatomical entity (GO: 0110165). This result also was consistent with the subcellular localization prediction of CPR5 proteins. To further identify the potential biological functions of *CPR5* in *Arabidopsis*, the protein-protein interaction analysis was performed and 10 potential interactors were detected (Figure 6B). Notably, several trichomes development related proteins (SIM, LGO, and GRL) directly interacted with CPR5, suggesting its regulatory role in trichomes formation. Moreover, CPR5 showed a highly closed relationship with several hormone response proteins, such as Salicylic acid signaling pathway regulators (SSI2, ACD6, EDS16, PAD4, and EDS1), Auxin signaling pathway regulator (AXR4).

### Genome-Wide Analysis of miRNA Targeting Gossypium *CPR5* Genes

**FIGURE 7:**
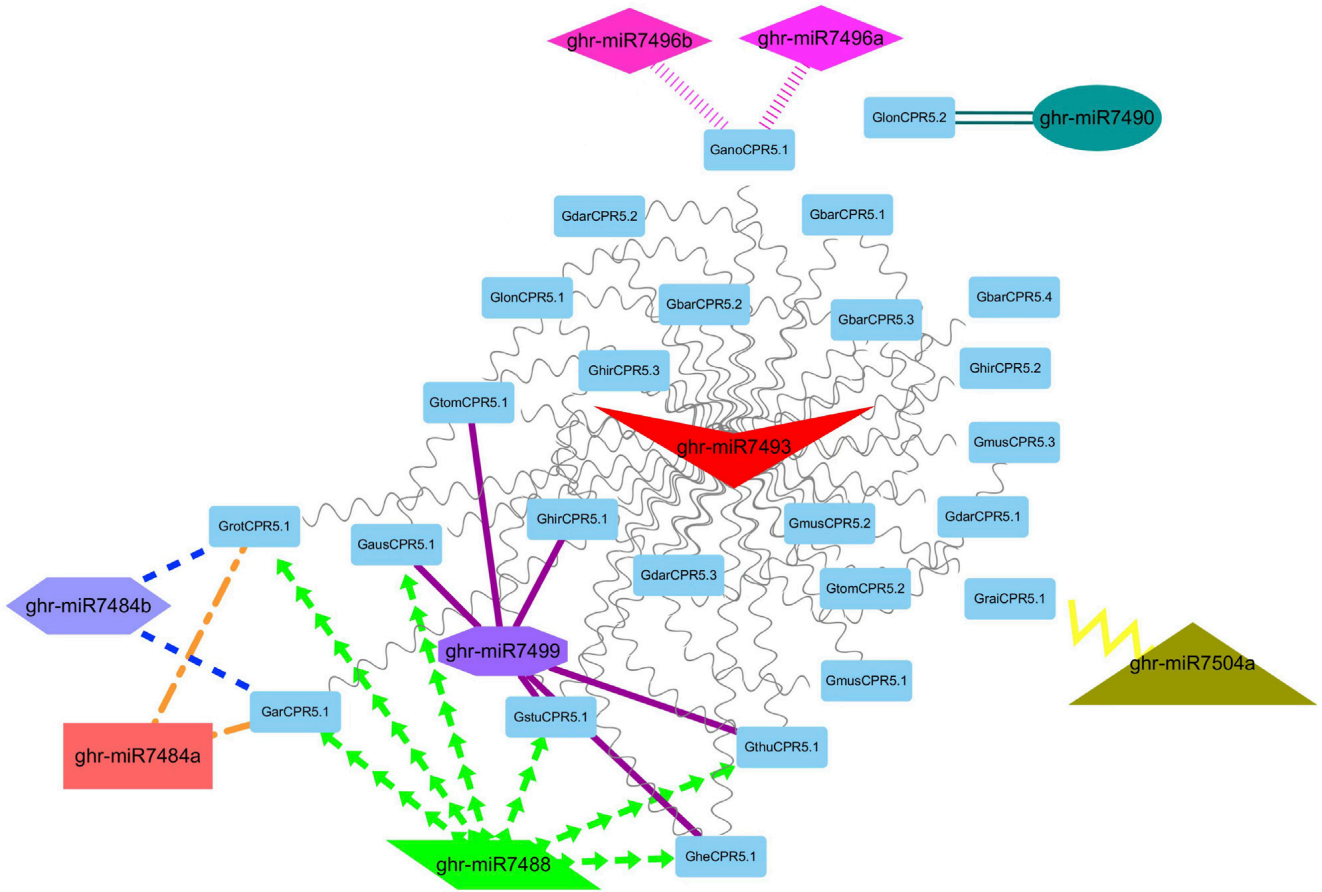
A network representation of the regulatory connections among the predicted miRNAs and *Gossypium CPR5* genes. Different colors highlight the interacting miRNAs. Similarly, different node colors and shapes clearly represent the interaction among miRNAs and Gossypium *CPR5* genes.

It has been reported previously that miRNAs dependent regulations have significant impacts on plant growth and regulation. Thus, to strengthen our understanding of the miRNAs associated with the regulation of *Gossypium CPR5* genes that are involved in the development of trichomes, we identified nine putative miRNAs targeting 25 *Gossypium CPR5* genes (Figure 7). The detailed information of the miRNA targeted sites is presented in Supplementary Table S3. We found that ghr-miR7490 interacts with one gene *GlonCPR5.2*, ghr-miR7496a, b interacts with *GanoCPR5.1*, ghr-miR7504a interacts with *GraiCPR5.1*, ghr-miR7484a,b interacts with *GarCPR5.1* and *GrotCPR5.1*, ghr-miR7499 interacts with *GtomCPR5.1*, *GausCPR5.1, GhirCPR5.1, GthuCPR5.1, GstuCPR5.1,* and *GheCPR5.1. Ghr-miR7488* interacts with *GrotCPR5.1, GausCPR5.1, GarCPR5.1, GthuCPR5.1, GstuCPR5.1,* and *GheCPR5.1*. Whereas ghir-miR7493 interacts with all the *CPR5* genes except *GlonCPR5.2* (Figure 7; Supplementary Table S3).

### Transcriptome Profiling and Co-Expression Network Analysis for Hub Gene Identification

**FIGURE 8:**
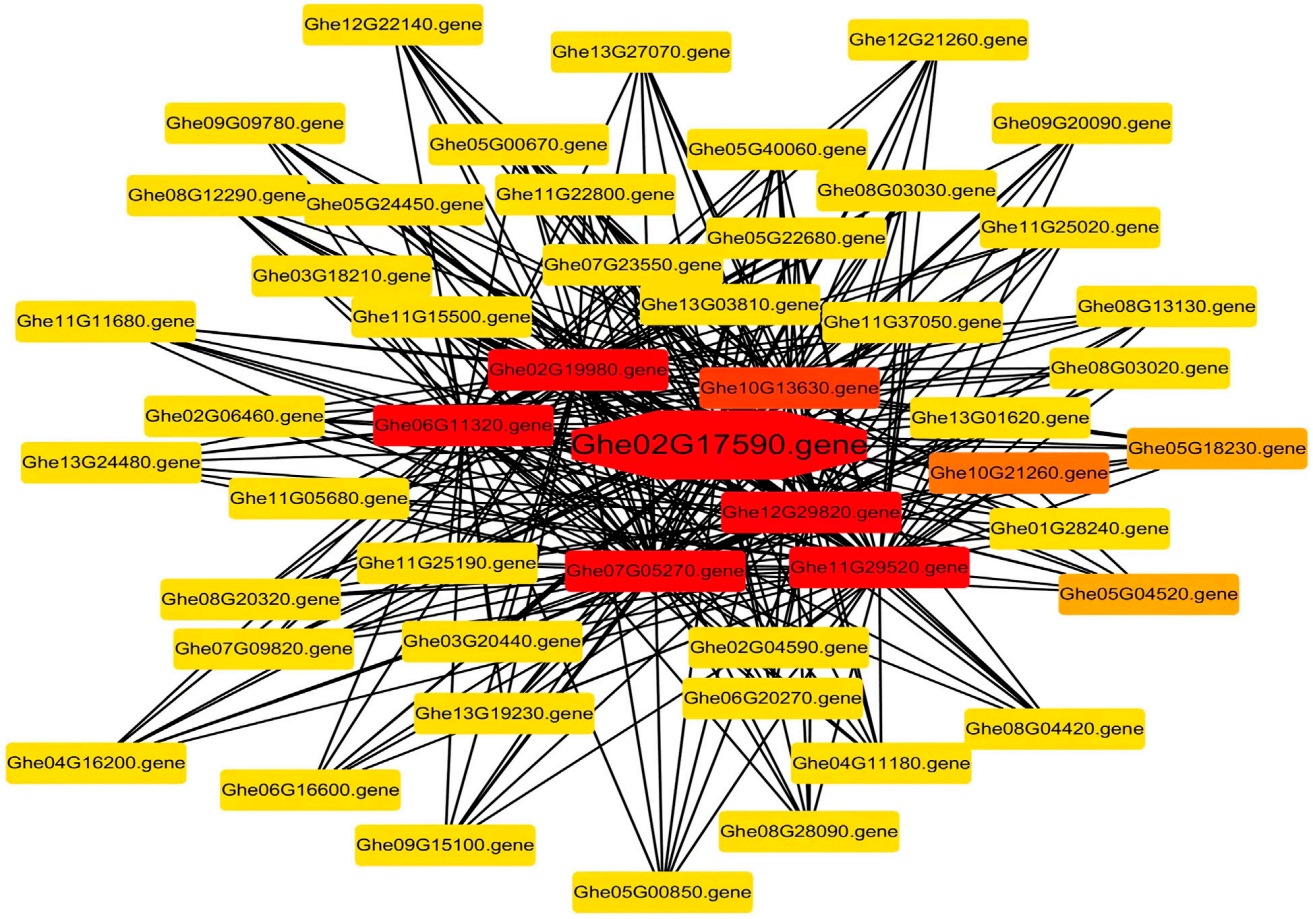
The Pearson correlation network analysis. Co-expression network of DEGs. The gene in the center of the network with red color and octagonal shape is represented as a hub gene.

In total, 9,673 differentially expressed genes were identified between *G. arboreum* with a high density of trichomes and *G. herbaceum* with barely any trichomes. A co-expression network analysis was performed by using the differentially expressed genes, to identify the hub genes linked to trichomes development and elongation (Figure 8). Correlation based relationships were plotted using a Pearson correlation coefficient greater than 0.99. A threshold level of >20 edges was considered as hub genes from the analysis. Owing to this, we found *Ghe02G17590* (*GheCPR5.1*) as a hub gene that might be involved in the trichome development.

### Tissue Specific Expression of *CPR5* Genes in Different Cotton Species

**FIGURE 9:**
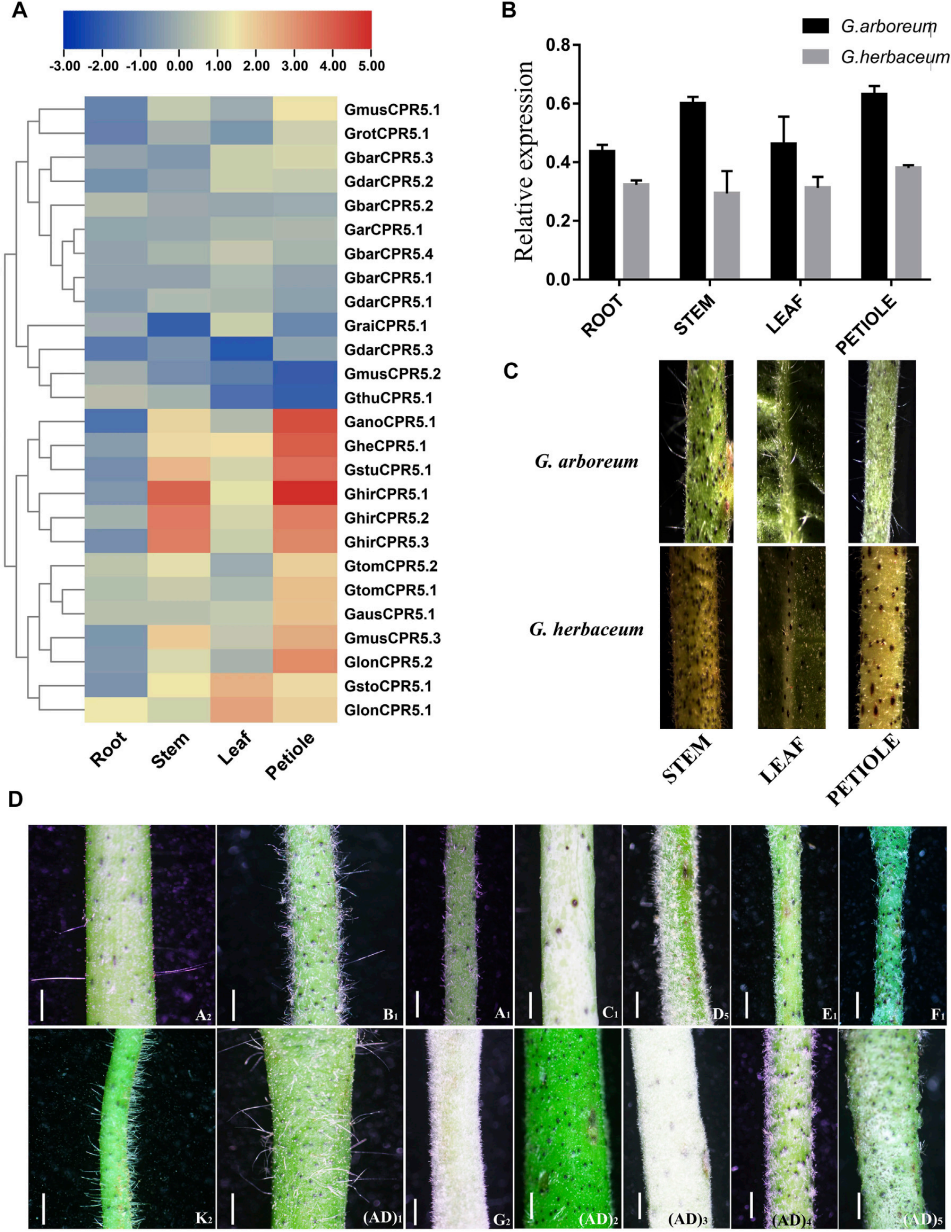
Expression of *CPR5* genes in different cotton species. **(A)-** Differential expression of all the *CPR5* genes in roots, stem, leaves and petioles. **(B)-** Expression analysis of *GheCPR5.1* in different tissues of *G. arboretum* and *G. herbaceum*. **(C)-** Density of trichomes in *G. arboretum* and *G. herbaceum.* **(D)-** Trichome density in all the Gossypium species used in this research work.

Expression of all identified *CPR5* genes was recorded in roots, stems, leaves and petioles of different cotton species (Figure 9A). Results suggested that higher expressions were recorded in the petioles as compared to the other tissues. Moreover, we noticed that the expression of *GheCPR5.1* was higher in the cotton species with trichomes.

### Expression Analysis of *GheCPR5.1* in Different Tissues of *G. arboreum* and *G. herbaceum*

RT-qPCR was performed to check the expression patterns of *GheCPR5.1* which was identified as a hub gene in the co-expression network analysis (Figure 9B). Expression was recorded in different tissues, i.e., of root, stem, leaves, and petiole of *G. arboreum* and *G. herbaceum*. We also performed the RT-qPCR to validate the expression of our candidate gene and the authenticity of RNA-seq data. *G. arboreum* with trichomes have higher expressions of *GheCPR5.1*in all the tissues, specifically petiole, as compared to *G. herbaceum* which has no trichomes. The RT-qPCR data was consistent with the RNA-seq data.

### Virus Induced Gene Silencing of *GheCPR5.1* for its Potential Role in Trichome Development and Elongation

**FIGURE 10:**
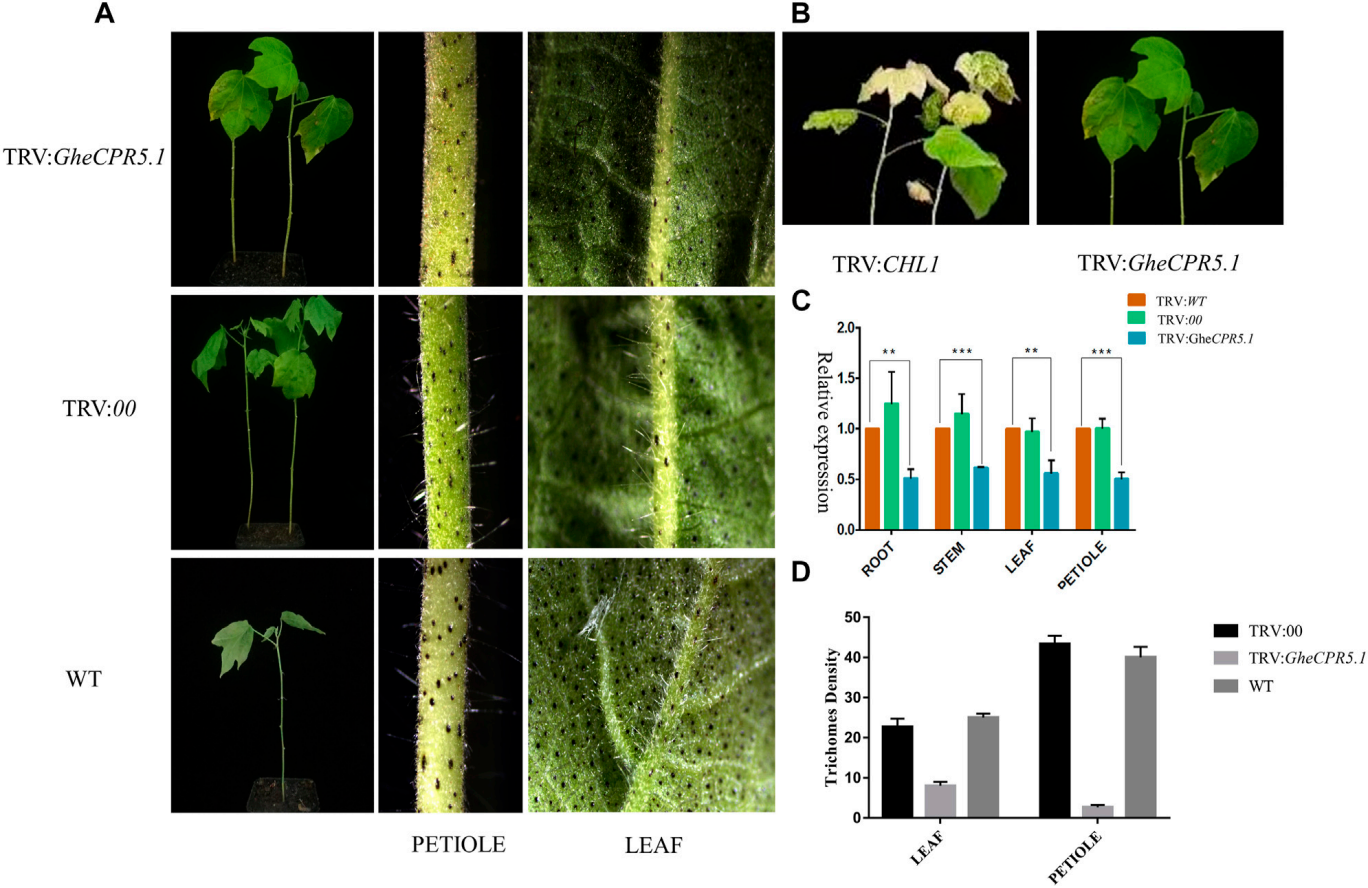
Virus induced gene silencing to elucidate the role of *GheCPR5.1* in trichome development and elongation. **(A)-** Visual images of trichomes after gene silencing. **(B)-** The blenching phenotypes were observed on TRV: *GheCPR5.1* plants. **(C)-** Relative expression level of *GheCPR5.1* in root stem and leaves of silences plants. **(D)-** Trichome density in the leaves and petioles after *GheCPR5.1* silencing in cotton.

The VIGS assay was performed to investigate the *GheCPR5.1* function related with the trichome development and elongation. The VIGS vector TRV: *GheCPR5.1* was injected into *G. arboreum* (Dianya-10, the long trichome line), to generate the *GheCPR5.1*-silenced plants. Twenty days after inoculation, the blenching phenotypes were observed on TRV: *GheCPR5.1*. The expression level of *GheCPR5.1* in wild type, negative control plant TRV: 00, and receptor plants TRV: *GheCPR5.1* were detected by RT-qPCR. No significant variations were observed in the expression of the *GheCPR5.1* gene in WT and TRV: 00. The expression of *GheCPR5.1* in TRV: *GheCPR5.1* lines was significantly lower than that of wild type and TRV: 00, indicating the success of the VIGS experiment Figure 10. The *GheCPR5.1*-silenced Dianya-10 plants exhibited a significant low density of long trichomes, compared with wild type and TRV: 00. These data suggest that *GheCPR5.1* plays a key role in trichome elongation in cotton.

## Discussion

In recent years, trichomes have been proven to be important for their involvement in multiple development pathways in various plant species, such as *Arabidopsis*, soybean, tomato, and tobacco (Lam and Pedigo, 2001; Choi et al., 2012; Cox and Smith, 2019; Fumin Wang et al., 2021). The trichomes functions of disease response, biotic stress defense, and maintaining the normal plant growth process have been deeply studied (Zhou et al., 2011; Yang and Ye, 2013; Zhou et al., 2013; Papierowska et al., 2020). Based on those results, lots of functional genes and signaling response pathways involved in trichomes initiation and elongation had been reported (Zhou et al., 2013; Yuan et al., 2019; Yu et al., 2021; Yuan et al., 2021). *CPR5* was first isolated from an Arabidopsis disease response mutant line, and its function to non-normal trichomes development had been identified in 1997 (Bowling et al., 1997). Compared with a classical *WD40*-*MYB*-*bHLH* complex (Zhao et al., 2013; Zhao et al., 2019; Yang et al., 2021) and other genes involved in trichomes development (Zhang et al., 2005; Zhang et al., 2019; Yu et al., 2021), the mechanism of *CPR5* regulating trichomes was still not clear. Furthermore, the cell structure of trichomes and fiber had been proved to share most similarity in cotton (Wang et al., 2004; Yang and Ye, 2013). Few research efforts focused on the genes regulating both trichomes and fiber and their function to cotton development (Shangguan et al., 2008; Shan et al., 2014).

In past few years, more research had been done on *CPR5* gene function analysis, especially in Arabidopsis. Research demonstrated multiple *CPR5* roles in plant growth (Kirik et al., 2001; Gao et al., 2011), plant immunity (Faisal et al., 2020; Van Dingenen, 2022), and trichome development by using mutant lines constructing, gene overexpression, and yeast split ubiquitin assay (Aki et al., 2007; Campbell et al., 2019). Previous results provided basic information of *CPR5* gene location, function, and its regulatory factors, but did not suggest their roles in different plant species evolutionary processes. In this study, we first identified all 26 *CPR5s* in Gossypium species, including four cultivars and seven wild species. Previous studies showed that *CPR5* genes were identified in both monocotyledons and dicotyledons. There were few *CPR5s* genes and their number varies in various plant species, such as six in *Triticum aestivum*, three in *Brassica rapa*, two in *Populus trichocarpa* and one in *Vitis vinifera*. Our results suggested that the *CPR5* genes number is consistent with the genomes number of plant species. Generally, only one copy of the *CPR5* gene is distributed in a diploid plant species, which indicated that *CPR5* is a classical single copy gene.

Unlike the traditional method of only reserving the longest transcript sequence for each gene identified through blastP, in this study, we chose the differential splicing *CPR5* sequences for the following analysis. The differential splicing alterations had been proved to produce numerous protein structures and function variances from one gene (Reddy, 2007; Park et al., 2013; Rehman et al., 2021). In the current study, we had identified multiple protein isoforms from one or two CPR5 genes in some Gossypium species, *GhirCPR5.2/GhirCPR5.3,GbarCPR5.1/GbarCPR5.2/GbarCPR5.3, GmusCPR5.2/GmusCPR5.3, GdarCPR5.2/GdarCPR5.3, GlonCPR5.1/GlonCPR5.2*. Between different cotton species, the differential splicing sequences shared similarities among amino acid sequence length, subcellular location, and motifs distribution. For example, the amino acid sequence length of *GhirCPR5.3* and *GbarCPR5.2* both were 401 aa and *GmusCPR5.3* was 409 aa. The same results were also seen for the CDS lengths of the genes mentioned above. Meanwhile, the subcellular location suggested that all the genes were located in the nucleus. Combining the gene structure results, the genes located in the nucleus did not possess the motif 2, motif 6, motif 7, and motif 13, which were predicted as four transmembrane features. Previous study suggested that a truncated CPR5 protein could be located at the plant cell nucleus (Perazza et al., 2011), which is consistent with our results.

Many previous studies had indicated that the single copy gene could be used as a molecular marker in understanding the phylogenetic relationships of closely related species (Sudmant et al., 2010; Sun and Komatsuda, 2010; Ananda et al., 2021). In this study, we constructed an unrooted phylogenetic tree by using all 33 *CPR5* genes identified from *Arabidopsis thaliana*, *Oryza sativa*, *Vitis vinifera*, *Theobroma cacao*, and Gossypium species. In this phylogenetic tree, the *CPR5* gene of *Arabidopsis* was present in clade A, the *CPR5* genes of *Oryza sativa* were present in clade B. Furthermore, we found the *CPR5* genes of *Vitis vinifera*, *Theobroma cacao,* and Gossypium species were grouped in the same clade C. The general evolutionary relationship indicated that *Vitis vinifera* and *Theobroma cacao* show a closer relationship with *Gossypium* as compared with *Arabidopsis* and *Oryza* (Chen et al., 2016). These results were consistent with the phylogenetic analysis between the already mentioned four genera. As for the evolutionary relationship within the 26 Gossypium species that were displayed in our phylogenetic tree, the *CPR5* genes that were identified from *G. rotundifolium* (K genome) and *G. australe* (G genome) showed a closer relationship with Theobroma, which is one of closest genera to Gossypium (Udall et al., 2019). Previous studies on phylogenetic relationships within Gossypium species also supported out results (Tang et al., 2015; Conover et al., 2019). According to these studies, *G. herbaceum* was closer to *G. arboreum*, *G. barbadense* and *G. hirsutum* were closer to *G. darwinii., G. tomentosum,* and *G. mustelinum* (Applequist et al., 2001; Renny-Byfield et al., 2016). The same phenomenon was also found in our study that *GheCPR5.1* and *GarCPR5.1* were grouped in one branch and *GhirCPR5.3, GbarCPR5.4, GtomCPR5.1, GmusCPR5.1*, and *GdarCPR5.2* were grouped in closer branches. The *CPR5* genes which share similar structures and motifs were also grouped together.

In this study, we chose five Gossypium species to display the *CPR5* genes chromosome location and the result showed that only one copy of the *CPR5* gene was located on chromosome 2 (*G. arboreum* A02 and *G. raimondii* D02) for all two diploid cotton species. For tetraploid species, all *CPR5* genes were found to be located on chromosomes A02 and D03. Previous study suggested that the tetraploid cotton species were formed by a hybridization process between A genome species and D genome species (Gong et al., 2012; Renny-Byfield and Wendel, 2014). These results indicated that the *CPR5* genes number of tetraploid cotton was twice than the number in diploid cotton, resulting in a whole-genome duplication event during the cotton polyploidization process.

The cis-elements including enhancers and promoters have been had their regulating mechanism of gene expression and function proven (Shan et al., 2014; Zhao et al., 2018). By identifying the Cis-element gaining and losing, it was critical to provide the source of morphological evolution influenced by gene function diversity (Li et al., 2020; Mengarelli and Zanor, 2021). In this study, several cis-elements of *CPR5* genes were predicted in Gossypium species. The same cis-elements, TCA-element, and TATA-box motifs, showed their core role in regulating *CPR5* gene expression. The studies showed that TCA-element is essential to regulating the genes expression to exogenous salicylic acid response in *Oryza sativa* (Nakshatri and Chambon, 1994; Rai et al., 2004). As for TATA-box, the function to the control transcriptional modulation mediated by miRNA have been identified in plant species (Yang et al., 2017; Lee et al., 2020; Ramalingam et al., 2021). Current results showed that the same *CPR5* genes regulate different regulatory mechanisms by various cis-elements distribution in plant species. The salicylic acid response element was mostly found in *CPR5* genes of Gossypium species. The *CPR5* gene has had its function proven to modulate salicylic acid to regulate pant growth and stress responses (Jirage et al., 2001; Lemarié et al., 2015). Those results revealed that *CPR5* would be regulated by multiple hormones, especially the salicylic acid. Gene ontology (GO) enrichment analysis showed that the *CPR5* genes were largely related to biological regulation, developmental process, and multicellular organismal process Xiaojing Wang et al., 2021.

MicroRNAs (miRNAs), that are a group of single-stranded, non-coding micro RNAs, are involved in post-transcriptional gene regulation (Cui et al., 2020). Various miRNAs have been identified *via* genome-wide analysis that are involved in growth and development in cotton (Zhang et al., 2007; Kwak et al., 2009; Ayubov et al., 2019). The current study identified nine miRNAs belonging to different families (ghr-miR7493, ghr-miR7484a, ghr-miR7484b, ghr-miR7488, ghr-miR7490, ghr-miR7496a, ghr-miR7496b, ghr-miR7499, ghr-miR7504a) targeting 25 Gossypium *CPR5* genes (*GheCPR5.1, GarCPR5.1, GhirCPR5.1, GbarCPR5.2, GausCPR5.1, GbarCPR5.1, GtomCPR5.1, GbarCPR5.3, GdarCPR5.2, GmusCPR5.2, GmusCPR5.3, GmusCPR5.1, GstuCPR5.1, GdarCPR5.3, GdarCPR5.1, GrotCPR5.1, GthuCPR5.1, GlonCPR5.1, GanoCPR5.1, GlonCPR5.2, GhirCPR5.2, GbarCPR5.4, GtomCPR5.2, GhirCPR5.3, GraiCPR5.1*). Discussed miRNAs in the current study are all involved in the cotton plant growth and development and trichome development as reported earlier (Guan et al., 2014; Xue et al., 2014; Li and Zhang, 2016; Wang et al., 2017; Xiaojing Wang et al., 2021). These studies suggest that these Gossypium-miRNAs might play potential roles in plant growth and trichome development by modifying the transcript level of the *CPR5* genes in Cotton.

In the current research based on transcriptome analysis of *G. arboretum* with trichomes and *G. herbaceum* with no trichome and co-expression network analysis we found that *Ghe02G17590* is the hub gene. This gene is also a *CPR5* gene and it has higher expressions in *G. arboretum* with trichomes and *G. herbaceum* with no trichomes (Yonekura-Sakakibara and Saito, 2013).

Virus induced gene silencing of *Ghe02G17590* confirms that this might be the true candidate gene that is involved in the trichome development and elongation. Virus induced gene silencing is an important method to predict the function of a candidate gene and previously many studies on cotton have been published to confirm the role of candidate genes (Zhao et al., 2021).

Keeping in view the importance of *CPR5* genes in plant growth and development and its role in trichome development further functional characterizations are needed to understand the molecular and genetics mechanisms of trichome development in cotton.

## Conclusion

The *CPR5* genes have a significant impact on crop tolerance to biotic and abiotic stress. Currently, we performed the genome wide identification, transcriptome analysis, co-expression, and RT-qPCR profiling and proved that *CPR5* genes have potential roles in trichome development. The co-expression network analysis and RT-qPCR results showed that *GheCPR5.1* is the hub gene and is involved in trichome development. Virus induced gene silencing of *Ghe02G17590* confirms its potential role in trichome development and elongation. This gene might have a positive contribution in trichome development in *G. hirsutum*. The importance of *CPR5* genes in plant growth, development, and trichome formation is summarized in this study and more functional characterization of *GheCPR5.1* is needed for conclusive findings at molecular levels.

## Data Availability

The datasets presented in this study can be found in online repositories. The name of the repository and accession number can be found below: NCBI; PRJNA833579.
