# Supplementary figures and images for: Genome-Wide Identification and Characterization of CPR5 Genes in Gossypium Reveals Their Potential Role in Trichome Development
Source: Front Genet. 2022 Jun 8;13:921096. doi: 10.3389/fgene.2022.921096 (PMC9213653; doi:10.3389/fgene.2022.921096)

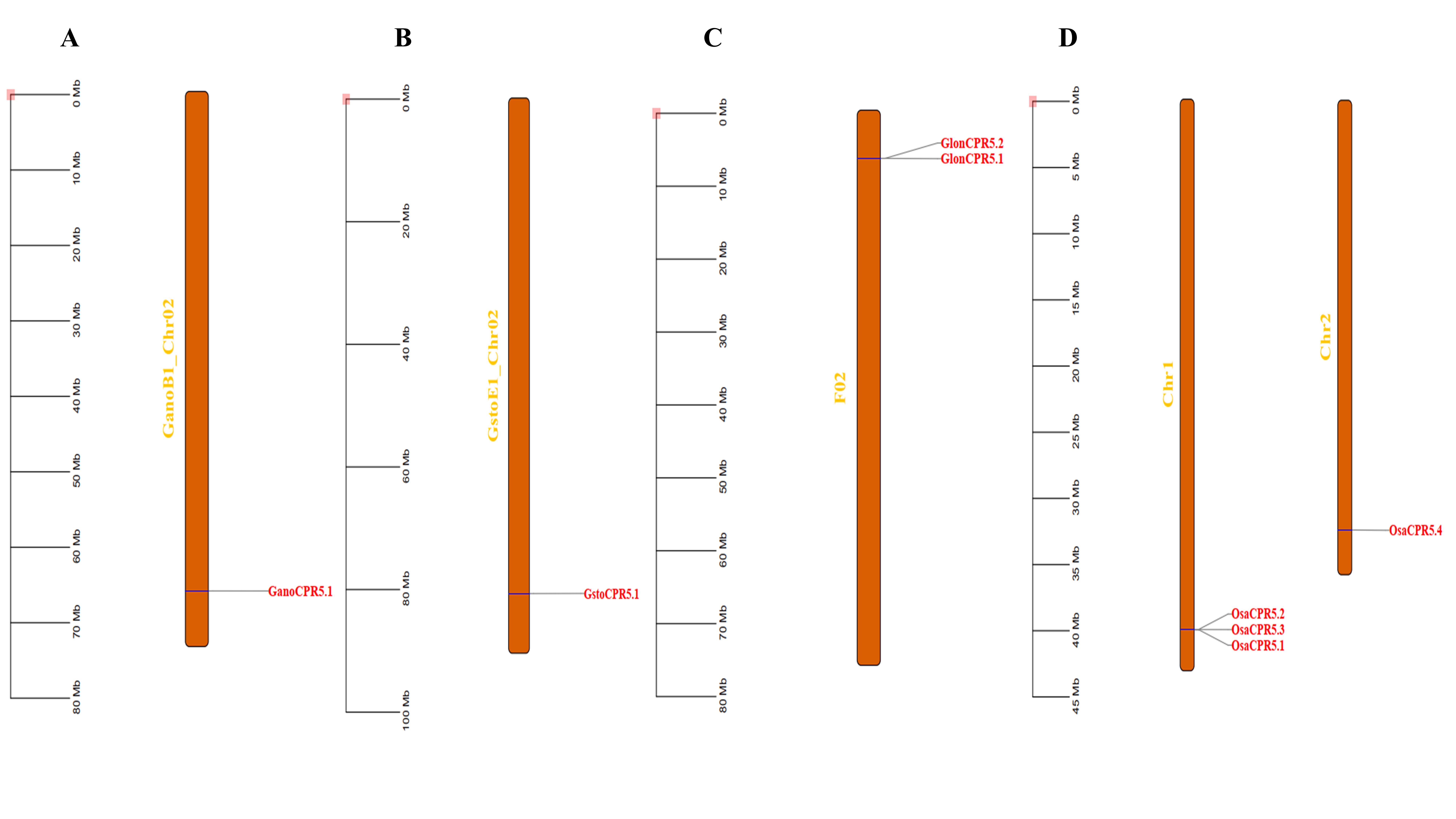

Supplement: Supplementary file 3 [file Image1.JPEG]
